# Decreased Local Specialization of Brain Structural Networks Associated with Cognitive Dysfuntion Revealed by Probabilistic Diffusion Tractography for Different Cerebral Small Vessel Disease Burdens

**DOI:** 10.1007/s12035-023-03597-0

**Published:** 2023-08-22

**Authors:** Mengmeng Feng, Hongwei Wen, Haotian Xin, Shengpei Wang, Yian Gao, Chaofan Sui, Changhu Liang, Lingfei Guo

**Affiliations:** 1 Department of Radiology, Shandong Provincial Hospital, Shandong University, Jing-wu Road No. 324, Jinan, Shandong 250021 China; 2 Key Laboratory of Cognition and Personality (Ministry of Education), Faculty of Psychology, Southwest University, Chongqing, 400715 China; 3 Research Center for Brain-inspired Intelligence, Institute of Automation, Chinese Academy of Sciences, ZhongGuanCun East Rd. 95 #, Beijing, 100190 China; 4 University of Chinese Academy of Sciences, Beijing, China; 5 Department of Radiology, Shandong Provincial Hospital Affiliated to Shandong First Medical university, Jing-wu Road No. 324, Jinan, Shandong 250021 China; 6 Key Laboratory of Endocrine Glucose & Lipids Metabolism and Brain Aging, Department of Radiology, Ministry of Education, Shandong Provincial Hospital Affiliated to Shandong First Medical University, Jinan, Shandong 250021 China

**Keywords:** Cerebral small vessel disease burden, Diffusion tensor imaging, probabilistic tractography, Structural network, Graph theory

## Abstract

To reveal the network-level structural disruptions associated with cognitive dysfunctions in different cerebral small vessel disease (CSVD) burdens, we used probabilistic diffusion tractography and graph theory to investigate the brain network topology in 67 patients with a severe CSVD burden (CSVD-s), 133 patients with a mild CSVD burden (CSVD-m) and 89 healthy controls. We used one-way analysis of covariance to assess the altered topological measures between groups, and then evaluated their Pearson correlation with cognitive parameters. Both the CSVD and control groups showed efficient small-world organization in white matter (WM) networks. However, compared with CSVD-m patients and controls, CSVD-s patients exhibited significantly decreased local efficiency, with partially reorganized hub distributions. For regional topology, CSVD-s patients showed significantly decreased nodal efficiency in the bilateral anterior cingulate gyrus, caudate nucleus, right opercular inferior frontal gyrus (IFGoperc), supplementary motor area (SMA), insula and left orbital superior frontal gyrus and angular gyrus. Intriguingly, global/local efficiency and nodal efficiency of the bilateral caudate nucleus, right IFGoperc, SMA and left angular gyrus showed significant correlations with cognitive parameters in the CSVD-s group, while only the left pallidum showed significant correlations with cognitive metrics in the CSVD-m group. In conclusion, the decreased local specialization of brain structural networks in patients with different CSVD burdens provides novel insights into understanding the brain structural alterations in relation to CSVD severity. Cognitive correlations with brain structural network efficiency suggest their potential use as neuroimaging biomarkers to assess the severity of CSVD.

**Supplementary Information:**

The online version contains supplementary material available at 10.1007/s12035-023-03597-0.

## Introduction

Cerebral small vessel disease (CSVD), one of the most common diseases in older adults, plays a vital role in dementia and stroke [1, 2]. The clinical manifestations of CSVD mainly include cognitive decline, gait disturbance, and psychiatric disorders. It can be visualized on neuroimaging scans as lacune, white matter hyperintensity (WMH), perivascular space (PVS), cerebral microbleed (CMB), recent small subcortical infarct, cortical superficial siderosis, brain atrophy, cortical cerebral microinfarct and summary small vessel disease score on brain MRI scans [3]. Previous studies have focused on the effect of a single CSVD feature and thus may have overlooked other features, but these neuroimaging features often occur simultaneously. Therefore, Pim Klarenbeek et al. proposed the concept of total CSVD burden, which integrates four previously mentioned CSVD features, to comprehensively evaluate the cumulative effects of various CSVD features on the brain and more completely estimate the severity of CSVD [4].

Recently, some studies have begun to pay attention to the CSVD burden. The MRI-based CSVD burden score improved diagnostic accuracy for predicting new onset of dementia after intracerebral hemorrhage [5]. Previous studies have shown that the CSVD burden was associated not only with the degree of damage to fiber tracts and reduced cortical thickness in a wide range of brain regions but also with gait/balance function and cognitive impairment [6–8]. Chen et al. paid attention to the multi-dimensional effects of CSVD burden on Parkinson’s disease (PD) and grouped PD patients according to the CSVD burden score (CSVD burden score 0–1 vs. 2–4). The results showed that motor dysfunction, cognitive impairment, depression, and anxiety were significantly worse in patients with severe CSVD than in those with mild CSVD [9]. A study on type 2 diabetes, divided patients into mild burden (0–1 points) group and moderate to severe burden (2–4 points) group based on the total CSVD burden score, demonstrated that the moderate to severe burden group was more likely than the mild burden group to have narrowed retinal arterioles, widened retinal venules, and lower arteriole-to-venule ratio [10]. These findings suggested that the CSVD burden affected the basic structure of gray and white matter in the brain, as well as the development and clinical manifestations of various diseases.

Despite these advances in research, the mechanism by which patients with different CSVD burdens exhibit clinical differences is still unclear. The relationship among different brain white matter structures should be taken seriously. The interregional interactions between anatomically separated structures could play important roles in processing complex clinical symptoms. Some studies on brain connectivity have suggested that human whole-brain structural networks can be reconstructed using diffusion tensor imaging (DTI) and probabilistic tractography [11, 12]. Graph theory, a powerful approach to characterize the interregional connective features within networks [13], can be used to obtain many important topological properties of brain structural networks, such as small-world topology, highly connected hubs and modularity [14], which exactly addresses the problem of interactions between different brain structures being neglected in some studies. Moreover, graph theory analysis can detect subtle changes in WM structure networks that may be overlooked by traditional techniques when the disease is not too serious [15], so as to better reveal the separation and integration status of the networks and the transmission of information.

Graph theory analysis has become increasingly popular in studying the CSVD burden and brain networks in CSVD patients. Previous studies have noted that an increasing burden of CSVD was associated with decreased nodal efficiency [6, 16] and small-worldness, reduced integration (increasing path length and lower global efficiency) and increased segregation (increasing clustering coefficient) of brain structural networks [6, 17]. Rutger Heinen et al. reconstructed structural brain networks using fiber tractography followed by graph theoretical analysis, assessed the relationship between total CSVD burden score, global network efficiency and cognition and showed that for every one-point increase in the CSVD burden score, global network efficiency decreased by 0.260 SD units [18]. These results provide new insights into the impact of CSVD burden on the structural network disruptions.

Building on previous works showing impaired structural connectivity networks in CSVD patients and the correlation between the CSVD burden and network parameters, we investigated the differences in the topological organization of the WM structural networks in patients with different CSVD burdens and controls through probabilistic diffusion tractography and graph theory analysis. Since CSVD patients often tend to experience cognitive decline which is affected by CSVD burden, the purpose of this study was to determine the relationship between the graph theoretical quantitative metrics and cognitive parameters of different CSVD burden groups.

## Materials and Methods

### Subjects

This was a cross-sectional study approved by the institutional review board of Shandong Provincial Hospital Affiliated to Shandong First Medical University. Between December 2018 and July 2022, 200 CSVD patients and 89 age-, sex- and education-matched healthy subjects were recruited in our study. Each participant voluntarily signed an informed consent form prior to the start of the study.

The inclusion criteria for CSVD patients comprised: (1) 40–75 years; (2) presence of lacune, WMH, PVS, CMB, recent small subcortical infarct, cortical superficial siderosis, brain atrophy, cortical cerebral microinfarct or summary small vessel disease score based on current MRI consensus standards [3].

The exclusion criteria included: (1) a history of stroke, brain trauma, epilepsy, brain tumors, intracerebral hemorrhage; (2) a history of major neurologic or psychiatric illnesses; (3) a history of alcohol or substance abuse; (4) a history of thrombolysis; (5) Alzheimer’s disease, Parkinson’s disease and other related diseases that cause cognitive impairment; (6) acute complications of type 2 diabetes and severe hypertension; (7) with significant hearing, vision and speech impairment; (8) unable to tolerate MRI or claustrophobia.

### MRI Acquisition and Evaluation

All subjects were imaged on a MAGNETOM Skyra 3.0 T MR scanner (Siemens Healthcare, Erlangen, Germany) using a product 32-channel head coil for signal reception. Diffusion weighted images (DWI) were acquired using a simultaneous multislice (SMS) accelerated single-shot echo planar imaging (EPI) sequence with the following parameters: repetition time (TR) = 3000 ms, echo time (TE) = 110 ms, 30 diffusion directions with b = 1700 s/mm^2^ and a single b = 0 s/mm^2^ acquisition, field of view (FOV) = 220 × 220 mm, matrix size = 110 × 110, 60 slices, and slice thickness = 2.2 mm with no intersection gap. 3D T1-weighted images were acquired using a magnetization-prepared rapid gradient echo (MPRAGE) sequence with the following parameters: TR / TE = 7.3 / 2.4 ms, inversion time (TI) = 900 ms, flip angle = 9°, FOV = 240 × 240 mm, matrix size = 256 × 256, 192 slices, and slice thickness = 0.9 mm with no gap. In addition, T2-weighted imaging, fluid-attenuated inversion recovery (FLAIR) imaging, and susceptibility-weighted imaging (SWI) were acquired to detect brain abnormalities. All participants were required to be awake and quietly breathing until the end of the scan.

Two experienced radiologists blinded to the clinical data independently evaluated CSVD imaging features on all MRI images based on the STRIVE criteria (STandards for ReportIng Vascular changes on Euroimaging) [3]. The CSVD burden is a pragmatic ordinal scale of 0–4 based on four MRI features of CSVD [4], their imaging performances and rating standards are included in the Supplementary materials. One point was awarded if ≥ 1 lacunes were present; one point was awarded if periventricular WMH reaches grade 3 or deep WMH reaches grade 2 or 3; one point was awarded if there were moderate to severe (grade 2–3) PVS in the basal ganglia; and one point was awarded if ≥ 1 CMB were present. In this study, CSVD patients were further grouped into those with a mild CSVD burden (CSVD-m; burden score 0–1; 133; age: 62.11 ± 6.88 years) and those with a severe CSVD burden (CSVD-s, burden score 2–4; 67; age: 64.16 ± 5.48 years). This stratification was consistent with the previous studies [9, 10].

### Cognitive Assessments

All participants underwent the Montreal Cognitive Assessment (MoCA) Beijing version (www.mocatest.org) which is a one-page 30-point test administered in 10 min [19]. The optimal cutoffs for detecting cognitive impairment was 13/14 points for illiterate individuals, 19/20 for individuals with 1–6 years of education, and 24/25 for individuals with 7 or more years of education [20]. In addition, a variety of executive functions, including flexibility, working memory and inhibition, were assessed. These tests included the following: the Rey auditory verbal learning test (AVLT) for assessing verbal memory abilities [21]; the symbol digit modalities test (SDMT) for evaluating attention and information processing speed [22]; the trail-making test (TMT) for evaluating attention, information processing speed, visual search and motor coordination [23]; and the Stroop color-word test (SCWT) [24]. The test implementer was professionally trained and qualified and had no knowledge of the subject grouping.

### Image Preprocessing

Following data acquisition, all the 3D T1-weighted images were manually reoriented to their respective anterior commissure–posterior commissure (AC-PC) plane using the statistical parametric mapping (SPM8, http://www.fil.ion.ucl.ac.uk/spm) toolbox. Then, we used the FMRIB software library (FSL v5.09, http://www.fmrib.ox.ac.uk/fsl) for diffusion image processing. Briefly, brain masks were created from the b0 image to remove nonbrain tissues using the Brain Extraction Tool (BET) in FSL. Second, eddy current distortions and motion artifacts were corrected by applying an affine alignment of each diffusion-weighted image to the b_0_ image and adjusting the gradient orientations to adapt to the slight rotations because of head movement parameters. Finally, the diffusion tensor model was fitted to each voxel using FMRIB’s diffusion toolbox (FDT v3.0), and the probabilistic distribution of fiber orientations from each voxel was estimated with a two-tensor model [25].

### Network Construction

**Fig. 1:**
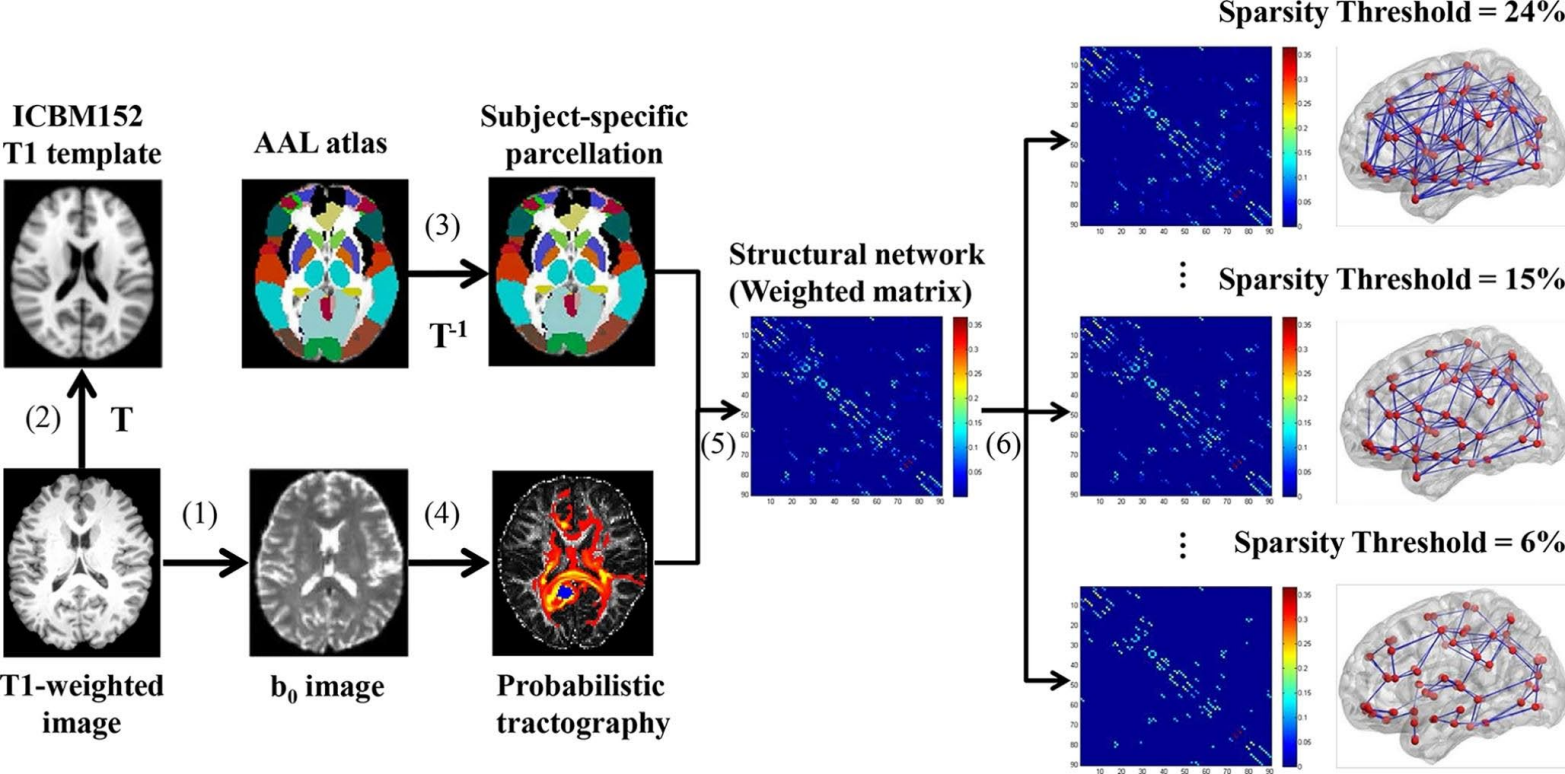
Flowchart of white matter structural network construction (1) Register the T1-weighted image to the b_0_ image in native DTI space for each subject by using an affine transformation. (2) The registered T1 images were then nonlinearly transformed to the ICBM152 T1 template in the MNI space, resulting in a nonlinear transformation (T). (3) Apply the inverse transformation (T^− 1^) to the AAL atlas (90 regions) in the MNI space, resulting in a subject-specific parcellation of node regions in native DTI space. (4) For each region, estimate the connectivity probability with other brain regions by using probabilistic diffusion tractography. (5) Construct the 90 × 90 weighted network for each subject by computing the connection probability between each pair of regions. (6) Threshold the individual matrix using a sparsity ranging from 6 to 24% with an interval of 1%. The visualization of the sparse networks is represented in lateral view

Network construction requires the following basic elements: nodes and connection edges, and the main steps are described in the following sections (detailed in Fig. 1).

Node definition: We defined the network nodes as in our previous study [12]. Briefly, individual T1-weighted images were coregistered to the b_0_ images in the DTI space by using an affine transformation (FLIRT tool in FSL). The registered T1 images were then nonlinearly transformed to the ICBM152 T1 template in MNI space by using FNIRT tool in FSL. The inverse transformations were used to warp the automated anatomical labeling (AAL) atlas [26] from MNI space to each native DTI space. Of note, the nearest-neighbor interpolation method was used to preserve discrete labeling values. Using this procedure, we obtained 90 cortical and subcortical regions (45 for each hemisphere), each representing a node in the network (Supplementary Table 1).

Connection definition: To define the connections between brain regions, we performed probabilistic tractography. First, we used the *bedpostx* tool in FSL to run Markov Chain Monte Carlo sampling to estimate distributions on diffusion parameters at each voxel, which allows us to model crossing fibers within each voxel of the brain. Second, we used the *probtrackx* tool in FSL to perform probabilistic tracking. Briefly, we repetitively performed 5000 samplings from the distributions of voxel-wise principal diffusion directions, each time computing a streamline through these local samples to generate a probabilistic streamline fiber. For a seed region, 5000×n streamline fibers were sampled; n is the number of voxels in the seed region. The number of streamline fibers passing through a given region divided by 5000×n was calculated as the connectivity probability from the seed region to the given region. The connection edges were defined as the above connectivity probability P_ij_ between regions i and j. In this study, each brain region based on the AAL atlas was selected as the seed region, and its connectivity probability to each of the other 89 regions was calculated. Thus, for each subject, a 90 × 90 probability weighted matrix was created to represent the constructed network. To remove spurious connections, the weakest connections under a given sparse threshold in the probability weighted matrix were discarded for each subject. To exclude the bias of a single sparse threshold, sparsity ranging from 6 to 24% with an interval of 1% was used as in previous studies [12, 27], and the network metrics were calculated at each threshold.

### Network Topological Analysis

To characterize the topological organization of WM structural networks, graph theoretical quantitative metrics were assessed at each threshold. The global properties include:

(1) The clustering coefficient (C_p_) of the network, which is the average nodal clustering coefficient (C_i_) over all nodes, and is computed as follows:

$${\text{C}}_{\text{i}}\text{=}\frac{\text{2}}{{\text{k}}_{\text{i}}{\text{(k}}_{\text{i}}\text{-1)}}\sum _{j,k}{\left(\stackrel{-}{{w}_{\text{ij}}} \stackrel{-}{{w}_{\text{jk}}} \stackrel{-}{{w}_{\text{ki}}}\right)}^{1/3}$$

where k_i_ is the degree of node i, and $\stackrel{-}{w}$ is the weight, which is scaled by the mean of all weights to control each subject’s cost at the same level.

(2) The shortest path length (L_p_) of network *G*, and is computed as follows:

$${\text{L}}_{\text{P}}\left(\text{G}\right)=\frac{1}{\text{N}(\text{N}-1)}\sum _{\text{i}\ne \text{j}\in \text{G}}{\text{L}}_{\text{i}\text{j}}$$

where L_ij_ is defined as the length of the path for node i and node j with the shortest length. For weighted networks, the length of each edge was assigned by computing the reciprocal of the edge weight (1/w_ij_).

(3–4) The normalized shortest path length λ = L_p_^real^/L_p_^rand^, and the normalized clustering coefficient γ = C_p_^real^/C_p_^rand^, in which L_p_^rand^ and C_p_^rand^ are the mean shortest path length and mean clustering coefficient of 100 matched random networks.

(5) The small-world index σ = λ/γ, and a real network would be considered to indicate a small world if γ > 1 and λ ≈ 1.

(6) The global efficiency (E_glob_) of network *G* measures the global efficiency of the parallel information transfer in network [28], which is computed as follows:

$${\text{E}}_{\text{g}\text{l}\text{o}\text{b}}\left(\text{G}\right)=\frac{1}{\text{N}(\text{N}-1)}\sum _{\text{i}\ne \text{j}\in \text{G}}\frac{1}{{\text{L}}_{\text{i}\text{j}}}$$

(7) The local efficiency (E_loc_) of network *G* reveals how much the network is fault tolerant and measures how efficient the communication is among the first neighbors of node i when it is removed, and is computed as follows:

$${\text{E}}_{\text{l}\text{o}\text{c}}\left(\text{G}\right)=\frac{1}{\text{N}}\sum _{\text{i}\in \text{G}}{\text{E}}_{\text{g}\text{l}\text{o}\text{b}}\left({\text{G}}_{\text{i}}\right)$$

where G_i_ denotes the subgraph composed of the nearest neighbors of node i.

(8) The nodal efficiency (E_nodal_), which measures the average shortest path length between a given node i and all of the other nodes in the network and is computed as follows:

$${\text{E}}_{\text{n}\text{o}\text{d}\text{a}\text{l}}\left(\text{i}\right)=\frac{1}{\text{N}-1}\sum _{\text{i}\ne \text{j}\in \text{G}}\frac{1}{{\text{L}}_{\text{i}\text{j}}}$$

Similar to previous studies, node i was considered a brain hub if E_nodal_(i) was at least one standard deviation (SD) greater than the average nodal efficiency of the network (i.e., E_nodal_(i) > mean + SD). We also calculated the area under the curve (AUC) for each network metric (global and local topological properties) to provide a summarized scalar independent of single threshold selection. The regional network measure and hub analyses were all conducted on the AUC value of E_nodal_. The graph theory analysis was implemented using a graph theoretical network analysis toolbox (GRETNA, http://www.nitrc.org/projects/gretna/) [29].

### Between-Group Statistical Comparison and Correlation Analysis

One-way analysis of variance (ANOVA) and least significant difference (LSD) post hoc multiple comparisons were used to assess demographic parameters and cognitive test scores among the three groups, and the chi-square test was used to analyze the sex proportions. For global and nodal network metrics, one-way analysis of covariance (ANCOVA) was performed to investigate differences among the three groups, controlling age, sex and education level as covariates, with LSD post hoc tests for pairwise comparisons. Once significant intergroup differences were identified in any nodal topological metrics, we further assessed the Pearson’s correlations between the nodal metrics and cognitive parameters for all groups using SPSS Version 24.0 (SPSS Inc, Chicago, IL, USA), and the significance level was set to p < 0.05 for all analyses.

## Results

### Demographic and Clinical Characteristics of the Subjects

The demographic and clinical characteristics of each group are summarized in Table 1. The CSVD-s group had significantly lower MoCA, the Rey auditory verbal learning test (AVLT), and the symbol digit modalities test (SDMT) scores and significantly higher the Stroop color-word test (SCWT) and the trail-making test (TMT) scores than the CSVD-m and control groups. In addition, the CSVD-m group had significantly lower SDMT scores than the control group. No significant differences were found in age, sex or education among the three groups.

**Table 1:** Demographic and clinical characteristics of CSVD patients and controls

Characteristic	CSVD-s	CSVD-m	HC	P value (ANOVA / χ^2^)	P value (post-hoc)		
					CSVD-s vs. HC	CSVD-s vs. CSVD-m	CSVD-m vs. HC
Sex	43 M / 24 F	70 M / 63 F	40 M / 49 F	0.058^χ2^	-	-	-
Age (y)	64.16 ± 5.48	62.11 ± 6.88	61.43 ± 9.54	0.071^a^	-	-	-
Hypertension	54 (80.6%)	67 (50.4%)	32 (36.0%)	< 0.001^χ2^	-	-	-
Diabetes mellitus	32 (47.7%)	67 (50.4%)	32 (36.0%)	0.096^χ2^	-	-	-
Hyperlipidemia	37 (55.2%)	59 (44.4%)	41 (46.1%)	0.333^χ2^	-	-	-
Smoking	17 (25.4%)	36 (27.1%)	15 (16.9%)	0.196^χ2^	-	-	-
Education (y)	11.13 ± 3.15	12.10 ± 3.29	12.38 ± 3.88	0.070^a^	-	-	-
MoCA	24.03 ± 2.97	25.26 ± 3.58	26.04 ± 3.79	0.003^a^	0.001	0.021	0.114
AVLT	54.76 ± 12.82	60.16 ± 13.14	63.11 ± 12.44	< 0.001^a^	< 0.001	0.006	0.100
SDMT	26.39 ± 11.24	31.55 ± 12.53	39.32 ± 14.05	< 0.001^a^	< 0.001	0.008	< 0.001
SCWT	175.44 ± 55.79	147.36 ± 43.95	137.12 ± 50.35	< 0.001^a^	< 0.001	< 0.001	0.133
TMT (B-A)	172.86 ± 94.78	127.47 ± 100.03	111.37 ± 93.14	< 0.001^a^	< 0.001	0.002	0.233
WMHs (1 point)	49 (73.1%)	6 (4.5%)	-	< 0.001^χ2^			
EPVSs (1 point)	59 (88.1%)	40 (30.1%)	-	< 0.001^χ2^			
CMBs (1 point)	44 (65.7%)	19 (14.3%)	-	< 0.001^χ2^			
Lacunes (1 point)	30 (44.8%)	3 (2.3%)	-	< 0.001^χ2^			

Abbreviations: CSVD = cerebral small vessel disease; MoCA = Montreal Cognitive Assessment; AVLT = sum of Rey auditory verbal learning test (N1-7); SDMT = symbol digit modalities test; SCWT = sum of Stroop color-word test (stroop1-3); TMT = the trail-making test; TMT (B-A) = the difference between TMT-B and TMT-A; WMHs = white matter hyperintensities; EPVSs = enlarged perivascular spaces; CMBs = cerebral microbleeds; CSVD-s = severe CSVD burden (score ≥ 2) group; CSVD-m = mild CSVD burden (score ≤ 1) group; HC = healthy controls; ^χ2^ = chi-square test; ^a^ =ANCOVA test. The last four rows are the number of subjects who scored one point for each CSVD burden features in the two CSVD subgroups

### Altered Global Properties of WM Networks in CSVD-s Patients

**Fig. 2:**
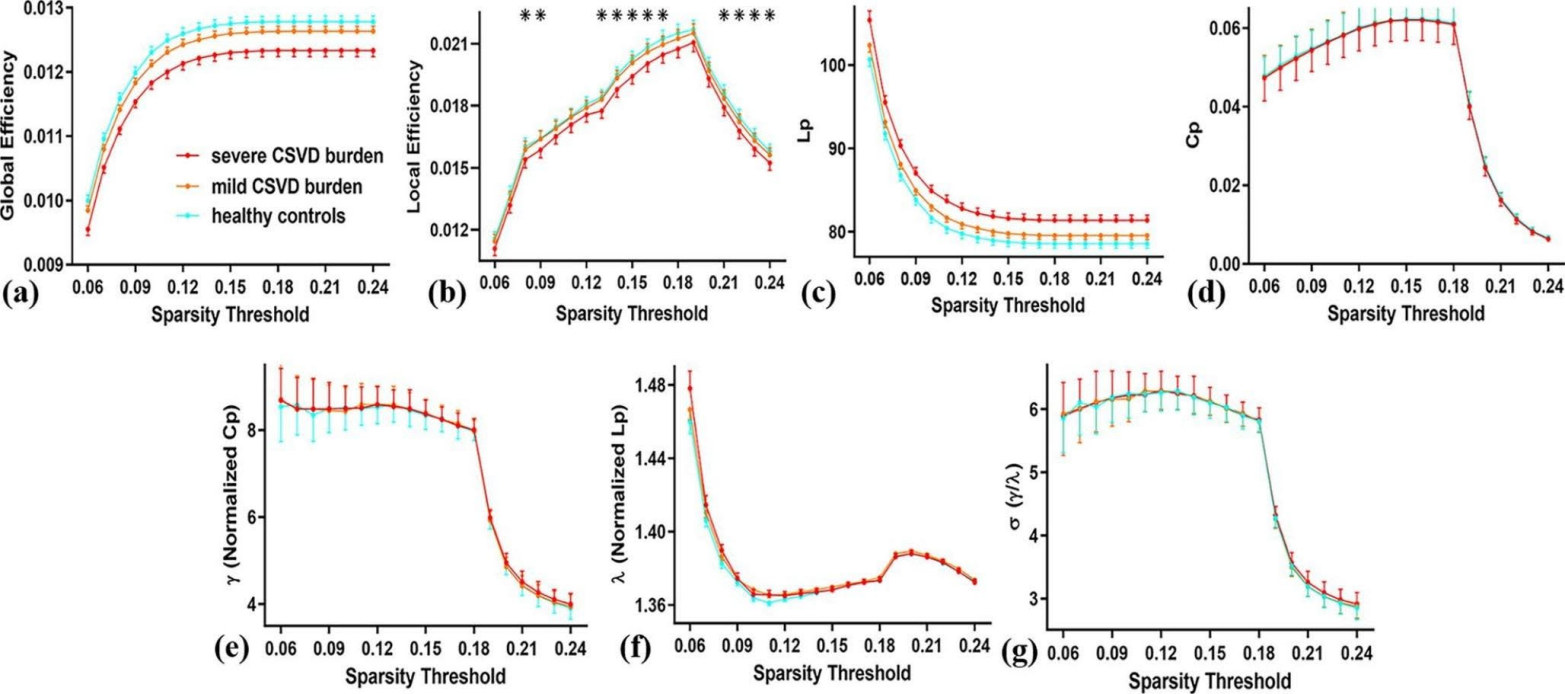
Group comparisons of global topological properties Data points marked with a star indicate the global property showing significant differences (p < 0.05, ANCOVA with LSD post-hoc test) in the CSVD-s group compared with the CSVD-m and control groups under a corresponding sparsity threshold. There was no difference between the CSVD-m and control groups

Over the whole sparsity range, the CSVD-s, CSVD-m and control groups all exhibited high-efficiency small-world topology characterized by γ > 1, λ ≈ 1 and σ = γ / λ > 1 (Fig. 2). Statistical comparisons (ANCOVA with LSD post hoc test) were performed to detect significant differences in global properties among the three groups. Compared with the CSVD-m and control groups, the CSVD-s group showed significantly (p < 0.05) decreased E_loc_ over a wide range of sparsity thresholds (Fig. 2b). No significant difference was found between the CSVD-m and control groups. Moreover, the CSVD-s group showed significantly decreased AUC values of E_loc_ compared with other groups (Table 2), indicating the consistency and robustness of significant alterations over sparsity thresholds. There was no significant difference in other global properties among groups.

**Table 2:** Group comparisons of AUC values of global properties

Global property (AUC value)	CSVD-s	CSVD-m	HC	P value (ANCOVA)	P value (post-hoc)		
					CSVD-s vs. HC	CSVD-s vs. CSVD-m	CSVD-m vs. HC
E_glob_ (×^e−2^)	1.19 ± 0.08	1.22 ± 0.09	1.24 ± 0.09	0.091^a^	-	-	-
E_loc_ (×^e−2^)	1.62 ± 0.10	1.67 ± 0.10	1.68 ± 0.11	0.035^a^	0.027	0.044	0.432
L_p_	84.64 ± 5.43	82.67 ± 6.18	81.55 ± 5.59	0.100^a^	-	-	-
C_p_ (×^e−2^)	4.49 ± 0.39	4.51 ± 0.39	4.53 ± 0.38	0.958^a^	-	-	-
γ	7.23 ± 0.23	7.21 ± 0.24	7.19 ± 0.18	0.704^a^	-	-	-
λ	1.38 ± 0.02	1.38 ± 0.02	1.38 ± 0.01	0.581^a^	-	-	-
σ	5.23 ± 0.15	5.22 ± 0.15	5.21 ± 0.13	0.835^a^	-	-	-

Abbreviations: AUC = area under the curve; E_glob_ = the global efficiency; E_loc_ = the local efficiency; L_p_ = the shortest path length; C_p_ = the clustering coefficient; γ = the normalized clustering coefficient; λ = the normalized shortest path length; σ = the small-world index

**Fig. 3:**
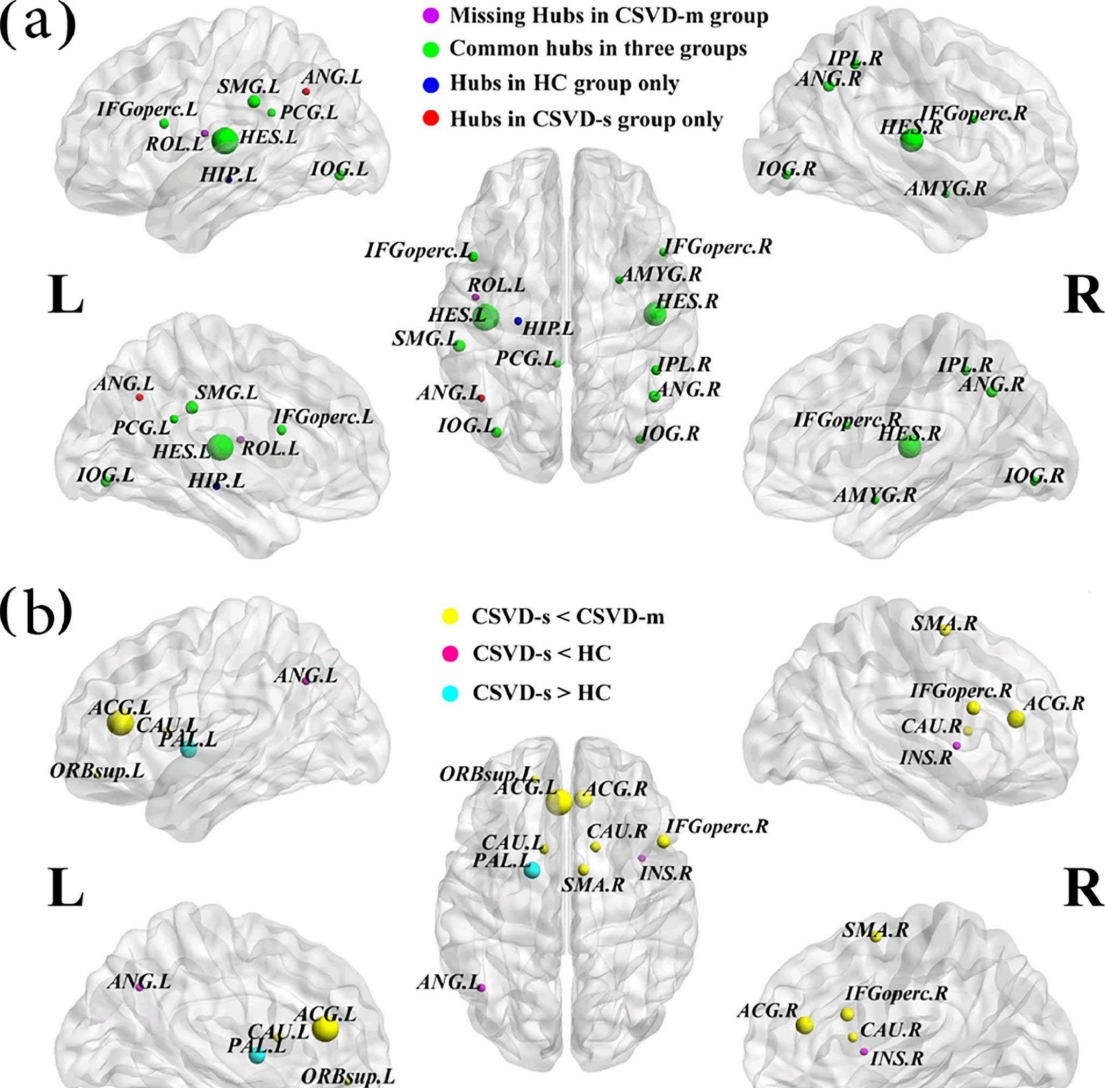
Hub region distributions in the WM structural networks and nodes with altered efficiency among the three groups (**a**) Hub nodes are displayed with different node sizes indicating their nodal efficiency values. (**b**) The CSVD-s group exhibited significantly increased nodal efficiency, and the scaled node sizes indicate the F values in the ANCOVA test. The brain graphs were visualized by using BrainNet Viewer software (http://www.nitrc.org/projects/bnv/). For the abbreviations of nodes, see Supplementary Table 1

### Partially Reorganized Hub Distributions of WM Networks in CSVD-s Patients

For each group, the nodes were considered brain hubs if their E_nodal_ was at least one SD greater than the average E_nodal_ of the network [12, 27]. We found partially reorganized hub distributions among groups with eleven common hub regions, including the bilateral inferior occipital gyrus (IOG), opercular inferior frontal gyrus (IFGoperc), Heschl gyrus, right inferior parietal gyrus, angular gyrus, amygdala and left supramarginal gyrus (SMG) and posterior cingulate gyrus. The CSVD-s and control groups each had an additional hub region in the left hippocampus and angular gyrus, respectively, and the CSVD-m group lacked the left Rolandic operculum as a hub region (Table 3; Fig. 3a).

**Table 3:** Hub regions of WM networks in both the CSVD and control groups

CSVD-s		CSVD-m		HC	
regions	E_nodal_ (×^e−2^)	regions	E_nodal_ (×^e−2^)	regions	E_nodal_ (×^e−2^)
IFGoperc.L	2.67	IFGoperc.L	2.86	IFGoperc.L	2.85
IFGoperc.R	2.48	IFGoperc.R	2.64	IFGoperc.R	2.73
PCG.L	2.44	PCG.L	2.52	PCG.L	2.56
AMYG.R	2.44	AMYG.R	2.52	AMYG.R	2.58
IOG.L	2.70	IOG.L	2.77	IOG.L	2.79
IOG.R	2.53	IOG.R	2.63	IOG.R	2.65
IPL.R	2.62	IPL.R	2.67	IPL.R	2.67
SMG.L	2.99	SMG.L	2.98	SMG.L	3.07
ANG.R	2.85	ANG.R	2.81	ANG.R	2.77
HES.L	4.73	HES.L	4.99	HES.L	5.07
HES.R	4.33	HES.R	4.48	HES.R	4.65
*ROL.L*	*2.38*			*ROL.L*	*2.50*
*HIP.L*	*2.41*			*ANG.L*	*2.53*

Abbreviations: WM = white matter; E_nodal_ = nodal efficiency. E_nodal_ represents the AUC value of nodal efficiency across thresholds; For the abbreviations of regions, see Supplementary Table 1

### Altered Regional Properties of WM Networks in CSVD-s Patients

Ten brain regions exhibiting significantly (ANCOVA, p < 0.05) altered E_nodal_ among the three groups were identified (Table 4). Furthermore, using the LSD post hoc test, pairwise intergroup differences were also identified. Compared with the CSVD-m or control groups, the CSVD-s group exhibited significantly decreased E_nodal_ in the bilateral anterior cingulate gyrus (ACG), caudate nucleus, right IFGoperc, supplementary motor area (SMA), insula and left orbital superior frontal gyrus (ORBsup) and angular gyrus (Table 4; Fig. 3b). Notably, compared with controls, the CSVD-m group showed significantly decreased E_nodal_ only in the left angular gyrus, while the CSVD-s group showed significantly increased E_nodal_ in the pallidum.

**Table 4:** Brain regions showing significantly altered nodal efficiency among the three groups for the WM networks

		E_nodal_ (×^e−2^)			p value (ANCOVA)	p value (post-hoc)		
Module	Region	CSVD-s	CSVD-m	HC		CSVD-s vs. HC	CSVD-s vs. CSVD-m	CSVD-m vs. HC
attention	ORBsup.L	1.64 ± 0.24	1.73 ± 0.25	1.67 ± 0.25	0.042	N.S.	0.007	N.S.
attention	IFGoperc.R	2.48 ± 0.27	2.64 ± 0.37	2.73 ± 0.29	0.015	0.002	0.042	N.S.
attention	ANG.L	2.37 ± 0.34	2.36 ± 0.37	2.53 ± 0.44	0.037	0.045	N.S.	0.012
sensory/motor	SMA.R	1.14 ± 0.23	1.26 ± 0.28	1.22 ± 0.27	0.021	N.S.	0.005	N.S.
sensory/motor	INS.R	1.84 ± 0.24	1.95 ± 0.32	2.02 ± 0.26	0.042	0.008	N.S.	N.S.
DMN	ACG.L	0.90 ± 0.13	0.97 ± 0.16	1.02 ± 0.19	0.002	0.001	0.015	N.S.
DMN	ACG.R	1.33 ± 0.22	1.44 ± 0.32	1.53 ± 0.33	0.007	0.006	0.041	N.S.
Subcortical	CAU.L	0.57 ± 0.16	0.68 ± 0.21	0.73 ± 0.22	0.030	0.022	0.027	N.S.
Subcortical	CAU.R	0.50 ± 0.09	0.58 ± 0.18	0.62 ± 0.16	0.027	0.003	0.035	N.S.
Subcortical	PAL.L	1.11 ± 0.79	0.88 ± 0.34	0.78 ± 0.26	0.008	0.007	N.S.	N.S.

Abbreviations: DMN = default mode network; N.S.= not significant. E_nodal_ represents the AUC values (mean ± SD) of the nodal efficiency of each group. For the abbreviations of regions, see Supplementary Table 1

### Altered Network Efficiency Related to Cognitive Performance in CSVD Patients

**Fig. 4:**
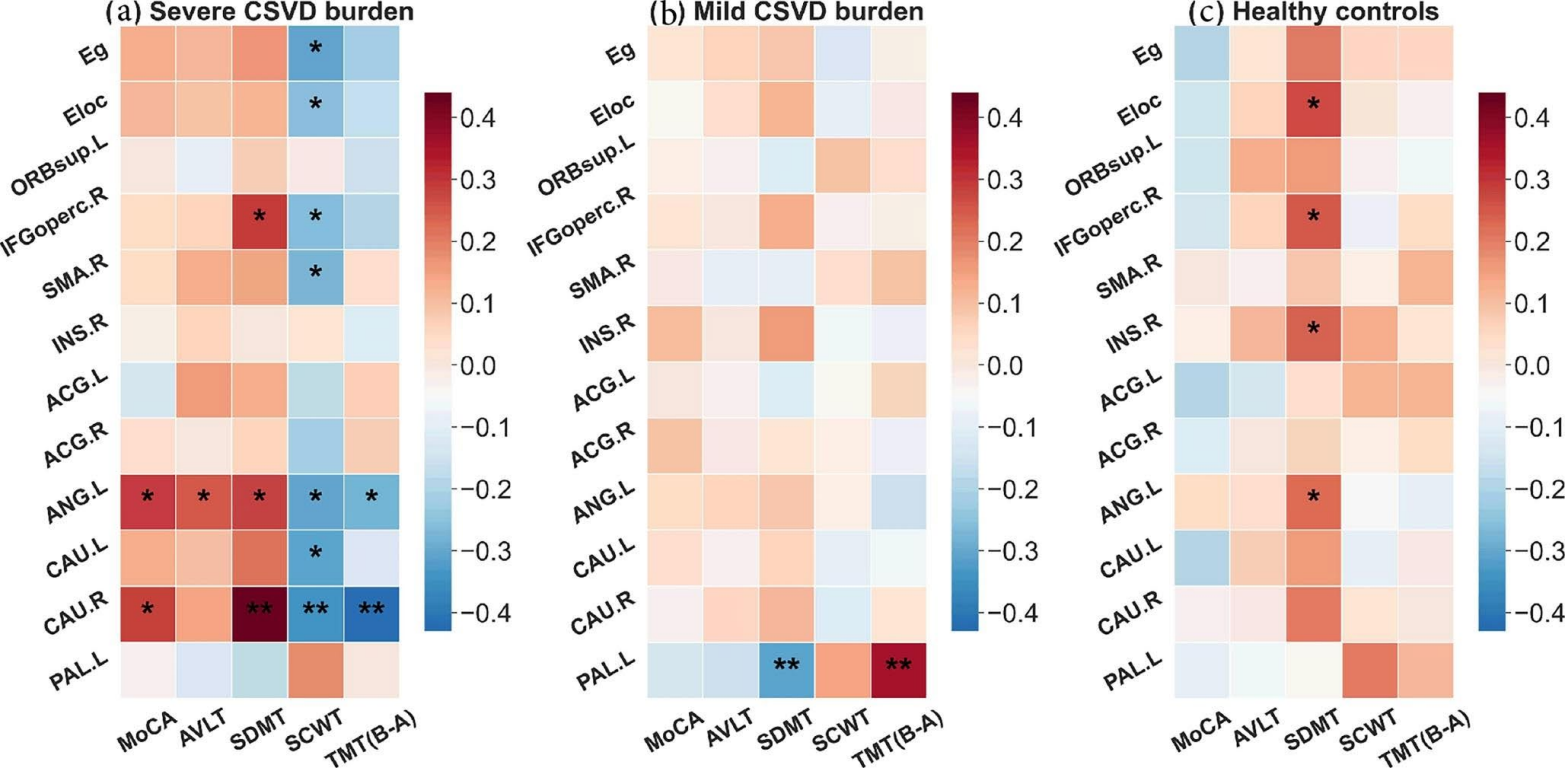
Pearson’s correlations between nodal efficiency and cognitive parameters in both the CSVD and control groups (**a**–**c**) Heat map of the correlation coefficient between nodal efficiency of disrupted regions and cognitive tests scores for the CSVD-s group, CSVD-m group and controls, respectively. *: p < 0.05, **: p < 0.01. For the CSVD-s group, network efficiency was significantly positively correlated with MoCA, AVLT, and SDMT scores and negatively correlated with SCWT and TMT (B-A) scores in multiple regions. For the abbreviations of nodes, see Supplementary Table 1

**Fig. 5:**
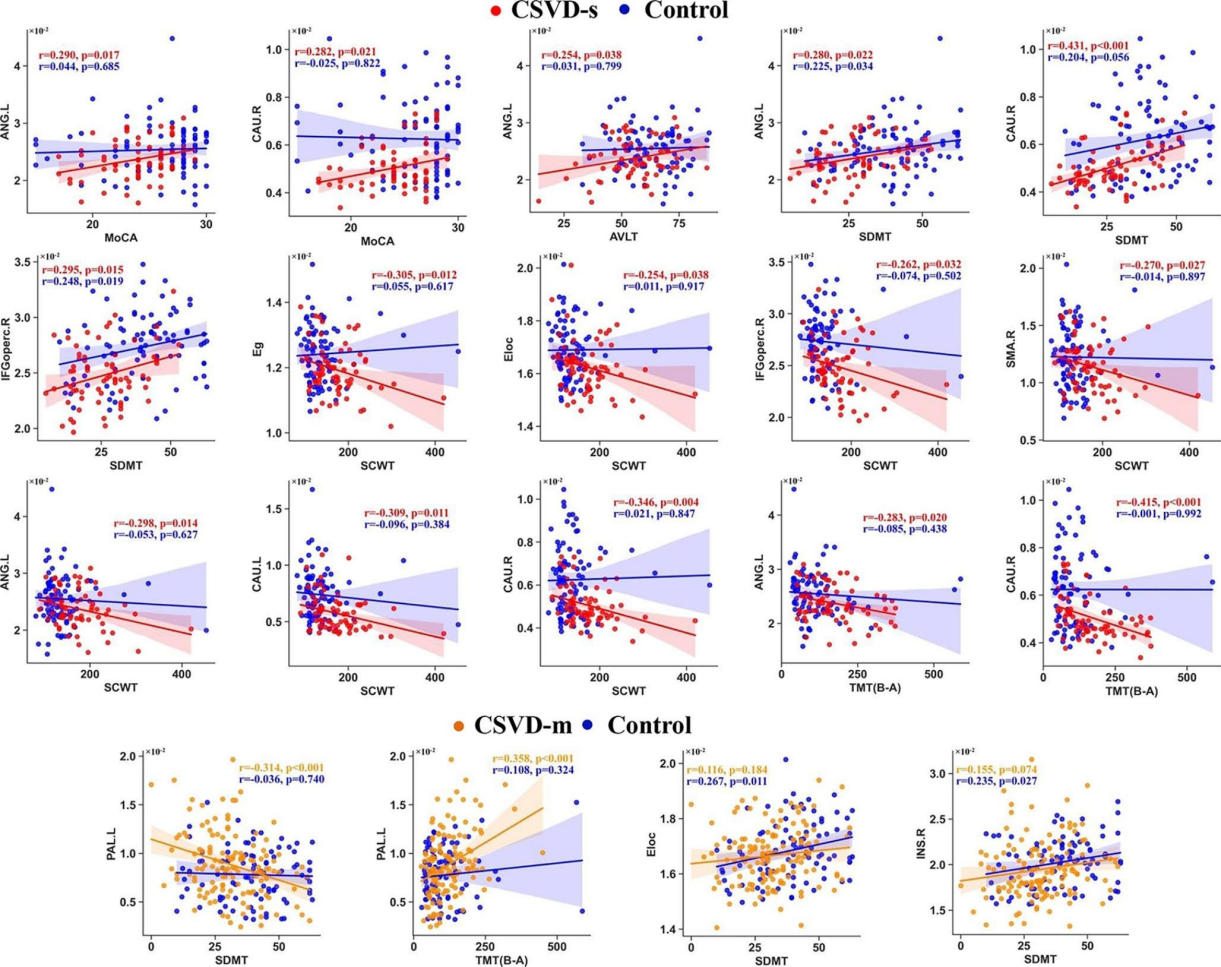
Scatter plots showing the significant (all p < 0.05, FDR corrected) Pearson’s correlations between network efficiency and cognitive parameters The red, orange and blue represent the CSVD-s, CSVD-m and control groups respectively. Linear regression lines with 95% confidence intervals for the best-fit line (shading area), as well as r (partial correlation coefficient) and p values, are provided. For the abbreviations of nodes, see Supplementary Table 1

For global/local efficiency and significantly altered nodal efficiencies among groups, participants Pearson’s correlations with cognitive parameters were calculated. Intriguingly, we observed significant correlations (p < 0.05, FDR corrected [30]) between network efficiencies and all cognitive parameters for the CSVD-s group (Fig. 4a), and the involved regions mainly included the bilateral caudate nucleus, right IFGoperc, SMA and left angular gyrus. Briefly, network efficiency in CSVD-s patients was significantly positively correlated with MoCA, AVLT and SDMT scores and negatively correlated with SCWT and TMT scores (Fig. 5). Moreover, only the left pallidum showed significant correlations with SDMT and TMT scores in the CSVD-m group (Fig. 4b).

## Discussion

In this study, the topological organization of WM structural networks was investigated using probabilistic diffusion tractography and graph theory. Compared with CSVD-m patients and healthy controls, CSVD-s patients exhibited significantly decreased E_loc_, which implied a disturbance in information exchange in the structural brain network of CSVD-s patients. Moreover, widespread decreased E_nodal_ in CSVD-s patients was found mainly in the cognitive functional regions. Intriguingly, significant correlations between network efficiency and cognitive performance scores were detected mainly in CSVD-s patients. These findings revealed the disruption of topological organization in structural networks in patients with CSVD, highlighted the importance of network analysis, and provided insights promoting a better understanding of the relationship between altered structural networks and cognitive deficits in patients with different CSVD burdens.

The human brain is understood to be a complex network with small-world properties, characterized by high local clustering and short path lengths, reflecting a balance between global integration and local segregation [31]. In this study, both CSVD patients and controls showed small-world properties of WM structural networks, which was consistent with previous studies of structural networks in CSVD patients [32]. compared with the CSVD-m and control groups, the CSVD-s group exhibited significantly decreased E_loc_, which means that the structural network has decreased efficiency in information processing and transfer in CSVD-s patients [33]. A considerable amount of research has shown that WM integrity is widely impaired in CSVD patients [34–36], and damage to WM leads to a breakdown in the structural and functional connections among specific brain regions [35, 37, 38], which cause reduced network efficiency [39]. Some researchers have suggested that disconnections between brain regions cause reduced network efficiency [39]. E_loc_ quantifies the resistance of a network to small-scale damage. Therefore, a reduced E_loc_ means that when one region is damaged or disconnected, its connections with linked regions are dramatically affected [40]. Our previous study on the functional brain networks revealed significantly decreased E_loc_ in CSVD patients with CMBs [41]. In addition, compared with the CSVD-m group, the CSVD-s group had more severe pathological changes, more diverse MRI features and more severe topological disruption. Therefore, compared with the control group, only the CSVD-s group changed significantly, while no significant change was observed in the CSVD-m group.

Apart from studying the global properties, we also investigated the nodal topology of structural networks. Hub regions occupy a central position and interact with many brain regions in the network, supporting their diverse functional roles across a broad range of tasks and widespread dynamic coupling within and across networks [42]. However, due to the high level of centrality of hub regions, these regions are susceptible to disconnection and dysfunction in brain diseases [42]. We defined the hub regions of brain structural networks according to the nodal efficiency of the network and found that the three groups had highly similar hub distributions, with hub regions mainly in the frontal gyri (bilateral IFGoperc), occipital gyri (bilateral IOG), parietal (right inferior parietal gyrus, angular gyrus and left SMG), temporal gyri (bilateral Heschl gyrus and right amygdala) and left posterior cingulate gyrus, supporting the view that the key regions of the structural network can tolerate developmental alterations and disease [12]. In addition, the CSVD-s and control groups each had an additional hub region in the left hippocampus and angular gyrus, respectively, and the CSVD-m group lacked the left Rolandic operculum as a hub region. This may be due to the changes in the brain structural networks during the course of the disease in CSVD patients, which affect the optimal paths of information transmission and ultimately lead to alterations in the hub distribution.

In this study, we also researched nodal efficiency and observed that the CSVD-s group had decreased E_nodal_ in nine brain regions, primarily located in subcortical, DMN, motor and attention functional modules [43], compared with the CSVD-m or control groups. Several studies have consistently shown that nodal efficiency decreased in brain regions associated with these functional modules in CSVD patients [16, 44, 45]. In addition, we found that the decrease in nodal efficiency was more severe in CSVD-s patients. Significantly decreased nodal efficiency in the CSVD-s group indicated that the ability to transmit information between this node and other nodes in the network decreased. With the progression of the disease, TNF-α expression in endothelial cells increases [46]. TNF-α increases blood brain barrier (BBB) permeability by inhibiting the expression of tight junction complexes [47]. Leakage of inflammatory substances into the brain through a disrupted BBB leads to central nervous system (CNS) inflammation [48]. In the presence of TNF-α, microglia can promote the release of glutamate from astrocytes, thereby enhancing the excitotoxicity of neurons [49]. In excitotoxicity, excessive release of glutamate leads to calcium overload in neurons, resulting in neuronal dysfunction, necrosis or apoptosis. Therefore, the destruction of the brain structure is more severe in CSVD-s patients, and it is not difficult to understand that the E_nodal_ reduction is more significant in this group. Intriguingly, the CSVD-s group showed increased E_nodal_ in the pallidum. We hypothesize that this is a compensatory mechanism of the brain for regions with reduced E_nodal_.

The CSVD burden is an important predictor for cognitive impairment in patients [50]. However, the role of brain structural network efficiency in the cognitive decline of CSVD patients has not yet been explored. We observed significant correlations between E_nodal_ and cognitive parameters in the CSVD-s group in the bilateral caudate nucleus, right IFGoperc, SMA and left angular gyrus. Importantly, E_nodal_ in the right caudate nucleus and left angular gyrus was significantly correlated with all five cognitive parameters. The caudate nucleus was suggested to be an important part of the brain’s learning and memory system and plays a role in stereotyped and repetitive functions [51]. The angular gyrus and its connectivity with other brain regions are involved in memory function to different degrees [52]. Moreover, the IFGoperc, a brain region whose function encompasses both social cognition and emotion, serves as both a sensory-cognitive integration area and a control node of the ventral attention network [53]. Damage to the SMA region can affect executive function/cognitive control [54]. Through the mediation of E_nodal_, these regions affect the cognitive function of the brain from multiple perspectives. We infer that the right caudate nucleus and left angular gyrus may be two key areas that are more correlated with the severity of cognition, which is important for the exploration of neurophysiological mechanisms in CSVD-s patients. Moreover, all network efficiencies were significantly correlated with the SCWT scores, indicating that CSVD-s patients were most susceptible to impairment of visual search speed, working memory and conflict monitoring ability. Based on Pearson’s correlation analysis, we speculate that only when the CSVD burden reaches a certain level will substantial damage to the brain structure occur, resulting in a linear decline in cognitive function. These results highlight the importance of investigating the correlation between network metrics and cognitive function in patients with different CSVD burdens, suggesting that early screening, diagnosis, detection and treatment of CSVD patients could prevent or delay cognitive decline.

Some limitations in this study should be considered. First, the use of a cross-sectional design implied that no conclusion about the temporality of alterations in CSVD burden, network properties, and cognition can be obtained. Second, this study only elaborated on disrupted WM structural networks, and follow-up studies should be conducted to combine structural and functional MR imaging to provide a comprehensive understanding with regard to the structure‒function coupling relationship. Third, although the CSVD burden was introduced in our study to compensate for the shortcomings of a single neuroimaging feature, we still need to investigate whether different scoring features have significant differences in brain structural changes under the same burden score.

## Conclusion

Using probabilistic diffusion tractography and graph theory, brain WM networks of CSVD are characterized by decreased local specialization, as well as widespread reduced network efficiency in cognitive functional regions. Patients with different CSVD burdens have segregated disruptions in WM network topology and associations with cognitive dysfunction. These findings expanded our understanding of brain structural alterations underlying CSVD and provided new theoretical bases for assessing the severity of CSVD.

### Electronic Supplementary Material

Below is the link to the electronic supplementary material.

Supplementary Material 1

## Data Availability

The datasets generated during and analysed during the current study are not publicly available due to [REASON(S) WHY DATA ARE NOT PUBLIC] but are available from the corresponding author on reasonable request.
