# Correction to: Decreased Local Specialization of Brain Structural Networks Associated with Cognitive Dysfuntion Revealed by Probabilistic Diffusion Tractography for Different Cerebral Small Vessel Disease Burdens

**DOI:** 10.1007/s12035-023-03647-7

**Published:** 2023-09-21

**Authors:** Mengmeng Feng, Hongwei Wen, Haotian Xin, Shengpei Wang, Yian Gao, Chaofan Sui, Changhu Liang, Lingfei Guo

**Affiliations:** 1 Department of Radiology, Shandong Provincial Hospital, Shandong University, Jing-wu Road No. 324, 250021 Jinan, Shandong China; 2 Key Laboratory of Cognition and Personality (Ministry of Education), Faculty of Psychology, Southwest University, 400715 Chongqing, China; 3 Research Center for Brain-inspired Intelligence, Institute of Automation, Chinese Academy of Sciences, ZhongGuanCun East Rd. 95 #, 100190 Beijing, China; 4 University of Chinese Academy of Sciences, Beijing, China; 5 Department of Radiology, Shandong Provincial Hospital Affiliated to Shandong First Medical university, Jing-wu Road No. 324, 250021 Jinan, Shandong China; 6 Key Laboratory of Endocrine Glucose & Lipids Metabolism and Brain Aging, Department of Radiology, Ministry of Education, Shandong Provincial Hospital Affiliated to Shandong First Medical University, 250021 Jinan, Shandong China

**Molecular Neurobiology**

10.1007/s12035-023-03597-0

The original version of this article unfortunately contained mistake in Abstract.

The Abstract currently published online is shorter than the original abstract, and has not been reviewed by the reviewers. With this, the Abstract has been updated.

The original article has been corrected.

