# Supplementary material for: Decreased Local Specialization of Brain Structural Networks Associated with Cognitive Dysfuntion Revealed by Probabilistic Diffusion Tractography for Different Cerebral Small Vessel Disease Burdens
Source: Mol Neurobiol. 2023 Aug 22;61(1):326–39. doi: 10.1007/s12035-023-03597-0 (PMC10791730; doi:10.1007/s12035-023-03597-0)
Supplement: Supplementary file 1 — Supplementary Material 1 [file 12035_2023_3597_MOESM1_ESM.docx]

2.2 Materials and Methods:

(1) lacune: Round or ovoid, subcortical, fluid filled (similar signal to cerebrospinal fluid (CSF)) cavity up to 15 mm in diameter; hyperintense on T2-weighted images, and hypointense on fluid-attenuated inversion recovery (FLAIR) and T1-weighted images [1].

(2) WMH: Signal abnormality of variable size in the white matter; hyperintense on T2-weighted and FLAIR images, and hypointense on T1-weighted imaging [1]. WMH of vascular origin are commonly categorized into periventricular WMH and deep WMH according to Fazekas Rating Scale，and two types of WMH have their own scoring standards [2]. Periventricular WMH: grade 0, absence; grade 1, “caps” or pencil-thin lining; grade 2, smooth “halo”; grade 3, irregular periventricular WMH extending into the deep white matter. Deep WMH: grade 0, absence; grade 1, punctate foci; grade 2, beginning confluence of foci; grade 3, large confluent areas.

(3) PVS: round, ovoid, or linear (depending on the slice direction) with a diameter commonly not exceeding 2 mm when imaged perpendicular to the course of the vessel, expansion of the fluid-filled space surrounding perforating arterioles; hyperintense on T2-weighted images, and hypointense on FLAIR and T1-weighted images [1]. PVS was graded based on the number of basal ganglia layers: grade 0, none; grade 1, <10; grade 2, 10-25; grade 3, >25 [3, 4].

(4) CMB: small round or ovoid, usually 2–5 mm or sometimes 10 mm; signal void with associated blooming artifact on T2* or other MRI sequences sensitive to susceptibility effects [1].

Supplementary Table 1 The 90 cortical and subcortical regions of interest defined in our study.

| Regions | Abbr. | Regions | Abbr. |
| --- | --- | --- | --- |
| Precentral gyrus | PreCG | Lingual gyrus | LING |
| Superior frontal gyrus, dorsolateral | SFGdor | Superior occipital gyrus | SOG |
| Superior frontal gyrus, orbital part | ORBsup | Middle occipital gyrus | MOG |
| Middle frontal gyrus | MFG | Inferior occipital gyrus | IOG |
| Middle frontal gyrus orbital part | ORBmid | Fusiform gyrus | FFG |
| Inferior frontal gyrus, opercular part | IFGoperc | Postcentral gyrus | PoCG |
| Inferior frontal gyrus, triangular part | IFGtriang | Superior parietal gyrus | SPG |
| Inferior frontal gyrus, orbital part | ORBinf | Inferior parietal, but supramarginal and angular gyri | IPL |
| Rolandic operculum | ROL | Supramarginal gyrus | SMG |
| Supplementary motor area | SMA | Angular gyrus | ANG |
| Olfactory cortex | OLF | Precuneus | PCUN |
| Superior frontal gyrus, medial | SFGmed | Paracentral lobule | PCL |
| Superior frontal gyrus, medial orbital | ORBsupmed | Caudate nucleus | CAU |
| Gyrus rectus | REC | Lenticular nucleus, putamen | PUT |
| Insula | INS | Lenticular nucleus, pallidum | PAL |
| Anterior cingulate and paracingulate gyri | ACG | Thalamus | THA |
| Median cingulate and paracingulate gyri | DCG | Heschl gyrus | HES |
| Posterior cingulate gyrus | PCG | Superior temporal gyrus | STG |
| Hippocampus | HIP | Temporal pole: superior temporal gyrus | TPOsup |
| Parahippocampal gyrus | PHG | Middle temporal gyrus | MTG |
| Amygdala | AMYG | Temporal pole, middle temporal gyrus | TPOmid |
| Calcarine fissure and surrounding cortex | CAL | Inferior temporal gyrus | ITG |
| Cuneus | CUN |  |  |

Abbreviations: Abbr. = abbreviation.

[1] Duering M, Biessels GJ, Brodtmann A, Chen C, Cordonnier C, de Leeuw FE, Debette S, et al. (2023) Neuroimaging standards for research into small vessel disease-advances since 2013. *Lancet Neurol* 22:602-618 <https://doi.org/10.1016/s1474-4422(23)00131-x>.

[2] Fazekas F, Chawluk JB, Alavi A, Hurtig HI, Zimmerman RA (1987) MR signal abnormalities at 1.5 T in Alzheimer's dementia and normal aging. *AJR Am J Roentgenol* 149:351-356 <https://doi.org/10.2214/ajr.149.2.351>.

[3] Doubal FN, MacLullich AM, Ferguson KJ, Dennis MS, Wardlaw JM (2010) Enlarged perivascular spaces on MRI are a feature of cerebral small vessel disease. *Stroke* 41:450-454 <https://doi.org/10.1161/strokeaha.109.564914>.

[4] Klarenbeek P, van Oostenbrugge RJ, Lodder J, Rouhl RP, Knottnerus IL, Staals J (2013) Higher ambulatory blood pressure relates to enlarged Virchow-Robin spaces in first-ever lacunar stroke patients. *J Neurol* 260:115-121 <https://doi.org/10.1007/s00415-012-6598-z>.
